# *MEF2C* is a potential prognostic biomarker for osteosarcoma

**DOI:** 10.1097/MD.0000000000044313

**Published:** 2025-09-05

**Authors:** Zhihui Zheng, Zhicheng Liao, Liang Pang, Zhouhengte Xu, Yibo Zhu, Pengcheng Jia, Qinglai Wang

**Affiliations:** a Department of Orthopedic Surgery, Wenzhou TCM Hospital of Zhejiang Chinese Medical University, Wenzhou, China.

**Keywords:** differentially expressed genes, expression quantitative trait loci, *MEF2C*, Mendelian randomization analysis, osteosarcoma

## Abstract

The purpose of this study was to investigate potential therapeutic targets for osteosarcoma (OS) and offer hints regarding genetic factors for OS treatment using a bioinformatics method. This study processed 3 OS datasets from the gene expression omnibus database using R software, screening for differentially expressed genes (DEGs). After enrichment analysis, based on expression quantitative trait loci data and the genome-wide association study data of OS, Mendelian randomization analysis was used to screen the genes closely related to OS disease, which intersect with DEGs to obtain co-expressed genes, validation datasets were employed to verify the results. Finally, the roles of co-expressed genes in OS were investigated through multiple bioinformatics methods. In this study, 269 DEGs (156 up-regulated genes and 113 down-regulated genes) were identified. Enrichment analyses indicated that DEGs play important roles in functions and pathways such as extracellular matrix organization, ossification, response to transforming growth factor beta, human T-cell leukemia virus 1 infection, and focal adhesion; Mendelian randomization analyses yielded 1 CEG that was significantly associated with OS (*MEF2C*). Validation analysis confirmed that *MEF2C* expression was significantly elevated in OS tissues, aligning with our differential expression results. Gene set enrichment analysis results indicated that *MEF2C* expression levels may correlate with alterations in biological activities pertinent to the regulation of skeletal system development and mineralization, as well as the enhancement and modulation of immune responses. Immune-cell-related analysis revealed a negative connection between *MEF2C* and dendritic cells activated. Clinical correlation analysis demonstrated a significant connection between *MEF2C* and patients with high-risk grade OS (*P* = .044). Survival analysis demonstrated *MEF2C*’s prognostic value for overall survival in lower-extremity OS (*P* = .02) and event-free survival (EFS) in both overall OS (*P* = .008) and lower-extremity subgroups (*P* = .007). Protein–protein interaction (PPI) network analysis showed that the effect of *MEF2C* on OS may be related to genes such as *APOE* and *SOX18*. The results of this study suggest that *MEF2C* is associated with an increased risk of OS and that *MEF2C* has the potential to be a prognostic biomarker for OS.

## 1. Introduction

Osteosarcoma (OS) is the most common primary malignant solid tumor of bone, accounting for approximately 56% of bone tumors.^[1,2]^ OS originates in the skeletal system and primarily affects children, adolescents, and young adults. It is most frequently diagnosed in individuals aged 15 to 19 years, with a median age of onset of 16 years.^[3,4]^ In recent years, the integration of surgical intervention and neoadjuvant chemotherapy has resulted in a notable enhancement in 5-year survival rates of OS patients.^[5,6]^ Approximately 68% of patients with limited-stage OS survive beyond 5 years. The prognosis for metastatic and recurrent OS is exceedingly unfavorable, with roughly 30% to 50% of patients advancing to recurrent illness.^[7–9]^ Although a variety of anticancer drugs have been used in the clinic, there is still no significant improvement in the treatment of metastatic and recurrent osteosarcoma.^[10]^ At present, targeted therapeutic research for osteosarcoma is still in the exploratory stage, and an in-depth study of the key mechanisms of its occurrence and development at the molecular level is required to provide a theoretical basis for the development of new therapeutic strategies.

Transcriptomics systematically identifies disease-related differentially expressed genes (DEGs) by analyzing transcriptome variations across samples, while simultaneously elucidating critical aspects of gene regulatory networks, tumor heterogeneity, and dynamic alterations in the immune microenvironment throughout the disease progression, thereby offering valuable insights for targeted drug development. Mendelian Randomization (MR) analysis is a method that uses genetic variation as an instrumental variable to analyze the causal relationship between exposure factors and disease.^[11,12]^ In this study, CEG associated with OS were identified through integrated transcriptomics and MR analysis. The specific functions and mechanisms of the co-expressed genes (CEGs) in the development of OS were further explored by various bioinformatics methods. This study reveals the important molecular mechanisms of OS, and lays a solid foundation for future therapeutic strategies.

## 2. Materials and methods

### 2.1. GEO data collection

In this study, gene expression microarray datasets relevant to OS are searched and downloaded from the gene expression omnibus (GEO) database. We utilized “Osteosarcoma” as the keyword, designated the organism as “Homo sapiens,” specified the entry type as “series,” and the study type as “expression profile by matrix.” We subsequently screened the datasets according to the following criteria: adherence to gene expression microarray data; possession of comprehensive gene expression data (raw or normalized); and inclusion of osteosarcoma tissues and normal control tissues within the samples of the dataset. Ultimately, we obtained 5 distinct gene expression microarray datasets. GSE16088, GSE42352, and GSE36001 served as training sets, whereas GSE12865 and GSE14359 functioned as validation sets (detailed information can be accessed from Table 1).

**Table 1:** The information of the 5 GEO datasets.

ID	N cases	N controls	Platforms	Experiment type	Last update date	Groups
GSE16088	14	6	GPL96	Array	August 10, 2018	Training set
GSE42352	84	15	GPL10295	Array	July 19, 2019	Training set
GSE36001	19	6	GPL6102	Array	November 04, 2009	Training set
GSE12865	12	2	GPL6244	Array	July 26, 2018	Validation set
GSE14359	18	2	GPL96	Array	August 10, 2018	Validation set

GEO = gene expression omnibus.

### 2.2. Methodology

#### 2.2.1. *Identification of DEGs*

The R software (version 4.3.3) was utilized to preprocess the 3 training group datasets, encompassing individual dataset correction as well as data normalization and standardization. To alleviate the influence of the batch effect, we employed the “prcomp” function to examine the principal components of the integrated data. Subsequently, the conventional Bayesian data analysis approach within the “limma” package was employed to screen DEGs.^[12]^ The filtering condition was set to an adjusted *P*-value <.05 and *|logFC*|>1, and generating volcano plots and heat maps to visualize the results.

#### 2.2.2. *GO/KEGG enrichment analysis*

The “clusterProfiler” package was used to analyze the gene ontology (GO) enrichment of DEGs, and explore the biological functions of these genes, including cellular component, molecular function (MF), and biological process (BP).^[13,14]^ Meanwhile, enrichment analysis was performed using the kyoto encyclopedia of genes and genomes to reveal the biological signaling pathways involved in DEGs.^[15]^ The screening criterion was an adjusted *P*-value of <.05.

#### 2.2.3. Exposure factors and outcome variables

In this study, we extracted the expression quantitative trait locus data provided by Võsa’s team from the IEU open GWAS database.^[16]^ We then screened single nucleotide polymorphisms (SNPs) as instrumental variables, which met the following criteria: significant correlation with the exposure factors (*P* < 5 × 10^−8^); removal of the effect of linkage disequilibrium (setting the parameter as *r*^2^ < 0.001, kb = 10,000); and exclusion of SNPs with an *F*-value of <10 (*F=β²/SE*², where *β* is the allelic effect value and *SE* is the standard error).

In addition, data from OS genome-wide association studies were extracted as outcome variables from the FinnGen database (version R12), which included 132 OS cases and 378,749 controls.

#### 2.2.4. MR analysis

MR analyses of instrumental and outcome variables were performed utilizing the “TwoSampleMR” package. Inverse variance weighting (IVW) served as the primary analytical method, while MR-Egger regression, weighted median, simple mode, and weighted mode were employed as secondary analytical methods. Subsequently, the results obtained from the MR analysis were screened, and the screening criteria included *P* < .05 for the IVW analysis results; the results from the above 5 analysis methods met the criterion of directional consistency (consistent direction of the odds ratios [ORs]); multiple hypothesis correction of the IVW results by the false discovery rate, with a corrected *P*-value of <.05; and there was no statistical significance in the test of multiple validity (*P* > .05). To ascertain the stability and reliability of the MR data, a heterogeneity test, multiple validity analyses, and a leave-one-out sensitivity analysis were conducted. The genes acquired from the screening were cross-referenced with DEGs to determine CEGs according to the orientation of *OR* values.

#### 2.2.5. Gene set enrichment analysis

GSEA can prioritize all genes from greatest to lowest according to the extent of differential expression. When specific biological functions or pathways exhibit an upregulation or down-regulation trend in gene expression, these pathways are typically considerably enriched at the upper or lower extremes of the ordered list, respectively.^[17]^ Therefore, in the present study, we further used the GSEA method, which aims to assess the degree of activity of specific genes concentrating on relevant functions and signaling pathways, with the significance judgment criterion set at *P* <.05.

#### 2.2.6. Analysis of immune-cell infiltration

To explore the probable function of CEG in immune regulation, the “CIBERSORT” package was employed to assess the infiltration features of 22 immune-cell types in OS samples and normal tissues, and to further investigate the association between CEG and these immune cells.^[18]^

#### 2.2.7. PPI network analysis

The STRING database (https://string-db.org) can be used to explore and analyze known and predicted interactions between proteins.^[19]^ Therefore, this study was conducted to analyze DEGs by protein–protein interaction network analysis through this database. The results were imported into Cytoscape (version 3.10.1), and the “Centiscape 2.2” plug-in was applied to determine the key compounds and visualize them.

#### 2.2.8. Clinical relevance analysis

In this study, we extracted the clinical information from the study of Lv et al (see Supplementary Material 1_Table S6, Supplemental Digital Content, https://links.lww.com/MD/P864) which contained data on gender, age, metastatic status, risk grade, and tumor site.^[20]^ After data collation, CEG were further analyzed to visualize whether there were significant differences between different clinical traits.

#### 2.2.9. Survival analysis

In this study, gene expression data and clinical information of 87 patients with osteosarcoma were obtained from the TARGET database (see Supplementary Material 1_Table S7, Supplemental Digital Content, https://links.lww.com/MD/P864). After data correction and processing, the survival analysis of CEGs was performed using the “survival” package to investigate the association between CEG and overall survival and Event-Free Survival (EFS) of patients with OS, and the association was considered to be significant at *P* <.05.

#### 2.2.10. Validation group analysis

The data of the 2 validation groups were processed and analyzed for differences using the same methods described above, and a total of 31 OS tissue samples and 4 normal tissue samples were included after merging. The results obtained were also compared with the results of MR analysis to verify whether the difference in the expression of CEG between OS tissues and normal tissues was statistically significant.

## 3. Results

### 3.1. Identification of DEG

**Figure 1.**
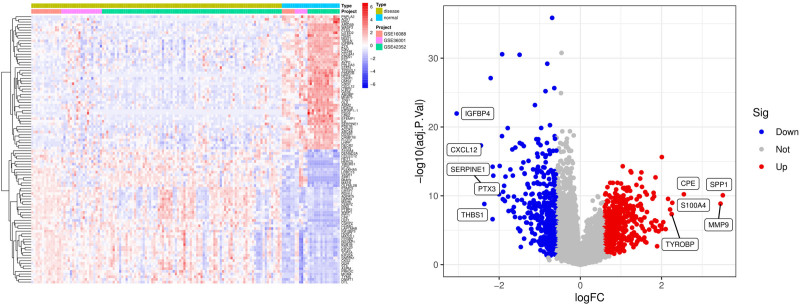
Differential gene expression heatmap and the volcano plot of DEGs with the 3 datasets. Disease: osteosarcoma tissue samples, normal: normal tissue samples. If the log fold change (*logFC*) is >1, it represents an up-regulated differentially expressed gene, which is the red part; if the *logFC* is less than −1, it represents a down-regulated differentially expressed gene, which is the blue part. (A) Differential gene expression heatmap; (B) the volcano plot of DEGs with the 3 datasets. DEGs = differentially expressed genes.

According to the screening criteria of DEGs, 269 DEGs were finally screened, including 156 up-regulated genes and 113 down-regulated genes (see Supplementary Material 1_Table S1, Supplemental Digital Content, https://links.lww.com/MD/P864). The heatmap shows the expression status of the top 50 up-regulated DEGs and the top 50 down-regulated DEGs and the volcano plots were used to demonstrate the expression characteristics of DEGs in the overall samples (Fig. 1).

### 3.2. GO/KEGG enrichment analysis

**Figure 2.**
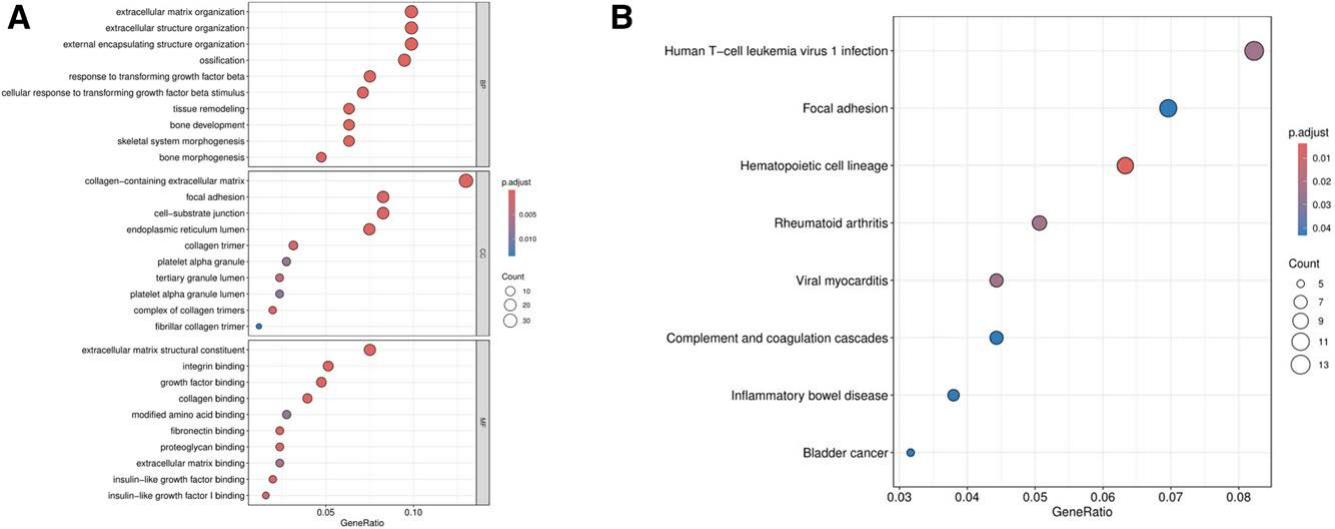
Results of the enrichment analysis of DEGs. The white circle indicates the number of genes enriched to the function or pathway by DEGs, the larger the shape of the circle, the more genes are enriched. (A) Results of GO enrichment analysis of DEGs; (B) results of KEGG enrichment analysis of DEGs. DEGs = differentially expressed genes, GO = gene ontology, KEGG = Kyoto encyclopedia of genes and genomes.

GO enrichment analysis showed that the BPs of DEGs were mainly enriched in extracellular matrix organization, extracellular structure organization, external encapsulating structure organization, tissue remodeling, and ossification, etc; the cytological components were mainly located in collagen-containing extracellular matrix, endoplasmic reticulum lumen, focal adhesion, cell-substrate junction, and complex of collagen trimers, etc. MF is mainly involved in extracellular matrix structural constituent, collagen binding, integrin binding, growth factor binding, and fibronectin binding, etc. The results of kyoto encyclopedia of genes and genomes enrichment analyses showed that DEGs were mainly enriched in pathways such as human T-cell leukemia virus 1 infection, focal adhesion, and hematopoietic cell lineage (see Supplementary Material 1_Table S2, Supplemental Digital Content, https://links.lww.com/MD/P864). In summary, the development of OS may be associated with functions and pathways related to extracellular matrix, bone morphogenesis, TGF-β signaling pathway and immune regulation. As shown in Figure 2.

### 3.3. MR analysis

**Figure 3.**
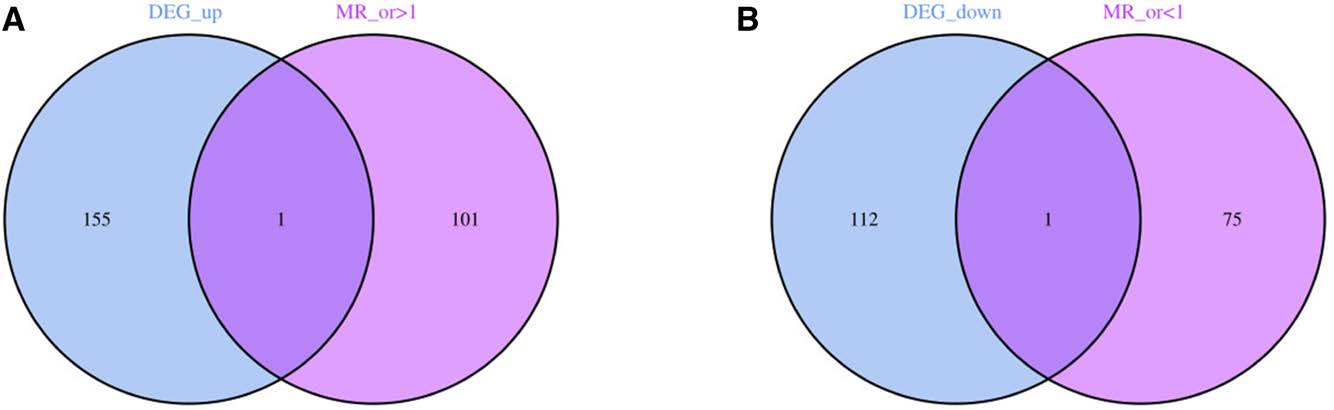
Venn plots. Two circles respectively represent the differential expression analysis results and Mendelian randomization analysis results of osteosarcoma. The overlapping part represents the co-expressed genes. (A) intersection of co-expressed up-regulated genes; (B) intersection of co-expressed down-regulated genes.

Based on the 3 major assumptions of the MR analysis and the *F*-value filtering criteria, we screened 26,513 SNPs as instrumental variables for inclusion in the subsequent analysis (see Supplementary Material 1_Table S3, Supplemental Digital Content, https://links.lww.com/MD/P864).^[12,21]^ MR analysis yielded 76 genes with *OR* <1 and 102 genes with *OR* >1 (see Supplementary Material 1_Table S4, Supplemental Digital Content, https://links.lww.com/MD/P864). After further intersection with DEGs, 2 CEGs, including 1 co-expression up-regulated gene (*MEF2C*) and 1 co-expression down-regulated gene (*TAGLN*), were finally identified. As shown in Figure 3.

**Figure 4.**
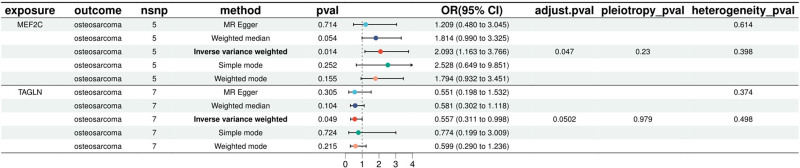
Forest plot of the CEGs. Results of MR analysis between co-expressed genes and osteosarcoma. OR = risk ratio, CEGs = co-expressed genes, CI = confidence interval, MR = Mendelian randomization, snp = single nucleotide polymorphism.

The results showed that there was a significant positive causal relationship between *MEF2C* and OS in the inverse variance weighted MR analysis of *MEF2C* (*OR* = 2.095; *95% CI*: [1.163 to 3.766]; *P* = .014) and *adjusted P*-value = .047. In addition to MR-Egger and weighted median, simple mode, and weighted models were used for further validation, respectively. It was found that all methods consistently suggested an increased risk of OS (*OR* > 1), and this directional consistency enhanced the reliability of the results. The results of the heterogeneity and multiplicity tests for *MEF2C* were both *P* > .05, indicating that there was no statistical significance and that there was no need to consider the effects of heterogeneity and multiplicity on the results. Leave-one-out sensitivity analyses showed that the effect values of each instrumental variable were close to the overall effect values (see Supplementary Material 1_Table S5, Supplemental Digital Content, https://links.lww.com/MD/P864). Detailed information on *MEF2C*, including scatter plots, forest plots, funnel plots, and leave-one-out sensitivity analyses, can be found in Supplementary Material 2, Supplemental Digital Content, https://links.lww.com/MD/P865. IVW results showed that *TAGLN* exhibited a significant negative causal relationship with OS (*OR* = 0.557; *95% CI*: [0.311–0.998]; *P* = .049). However, after correction, no significant difference was found between the 2 (adjusted *P*-value >.05), so the next analysis of the 2 was not performed in this study for the time being. As shown in Figure 4.

### 3.4. GSEA

**Figure 5.**
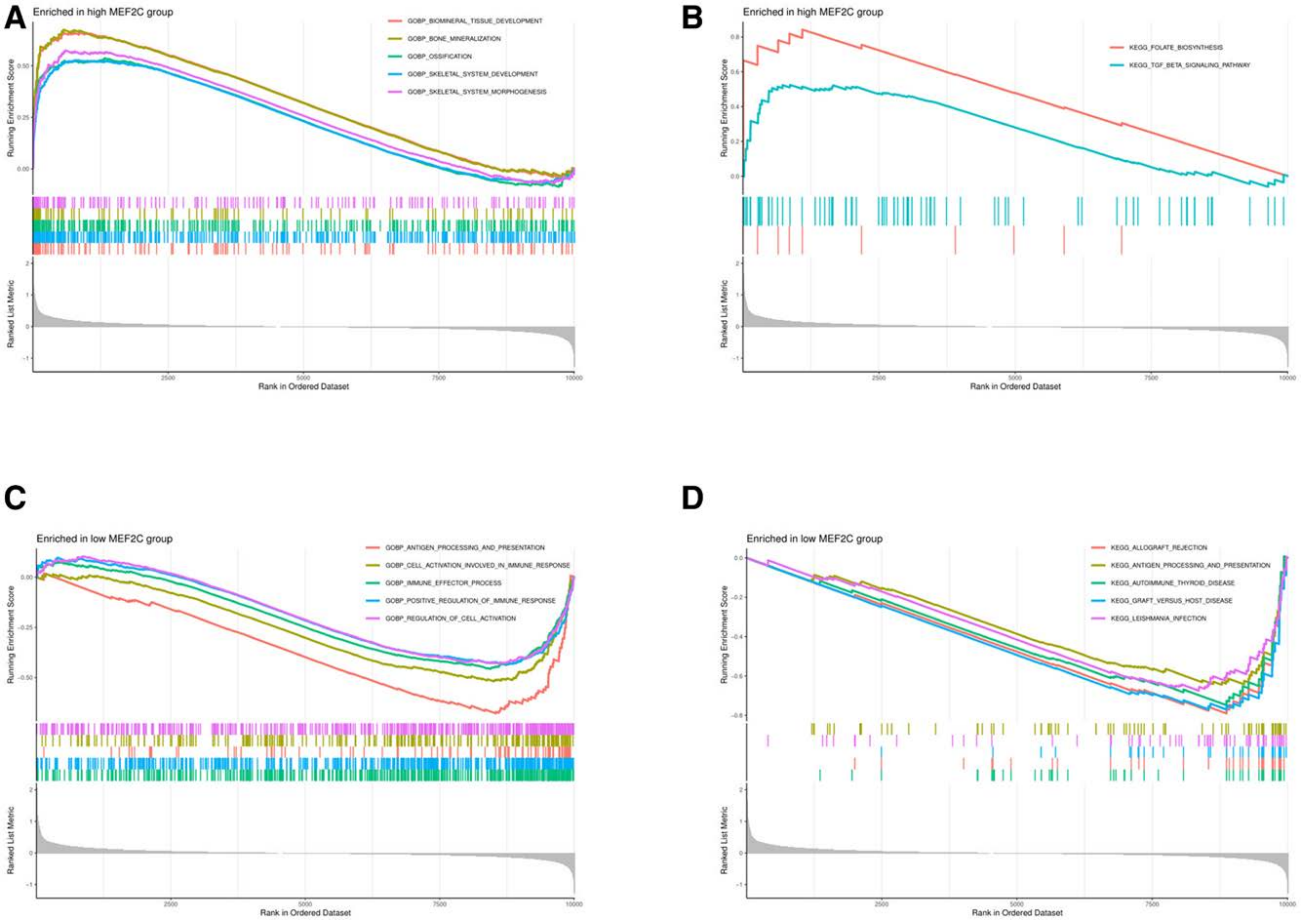
Gene set enrichment analysis of *MEF2C.* (A) functional enrichment analysis of *MEF2C* high-expression group; (B) pathway enrichment analysis of *MEF2C* high-expression group; (C) functional enrichment analysis of *MEF2C* low-expression group; (D) pathway enrichment analysis of *MEF2C* low-expression group.

GSEA results showed that the top 5 active biological functions in the *MEF2C* high-expression group were biomineral tissue development, bone mineralization, ossification, skeletal system development, and skeletal system morphogenesis; the top 2 active pathways were folate biosynthesis and the TGF-β signaling pathway; the top 5 active biological functions in the *MEF2C* low-expression group were antigen processing and presentation, cell activation involved in immune response, immune effector process, positive regulation of immune response, and regulation of cell activation; and the top 5 active pathways were allograft rejection, antigen processing and presentation, autoimmune thyroid disease, graft-host disease, and leishmania infection. In summary, the expression level of *MEF2C* may be related to the development of the skeletal system, immune effector process, and other changes in biological functional activities. As shown in Figure 5.

### 3.5. Immune-cell infiltration analysis

**Figure 6.**
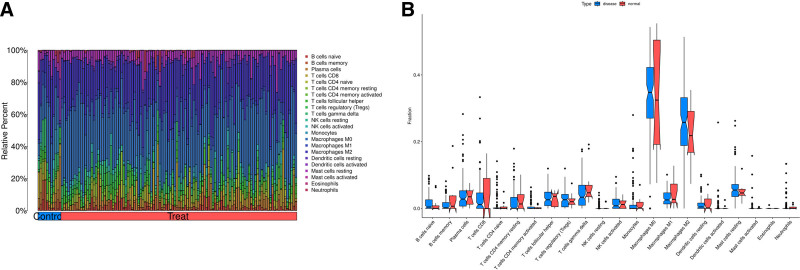
Analysis of immune cell infiltration in osteosarcoma. Disease: osteosarcoma group, normal: normal tissue group. (A) Stacked histograms of the proportion of immune cells in the osteosarcoma group versus the normal tissue group; (B) box-and-line plots comparing the infiltration levels of 22 immune cells in the osteosarcoma group versus the normal tissue group. *P <.05, **P <.01.

**Figure 7.**
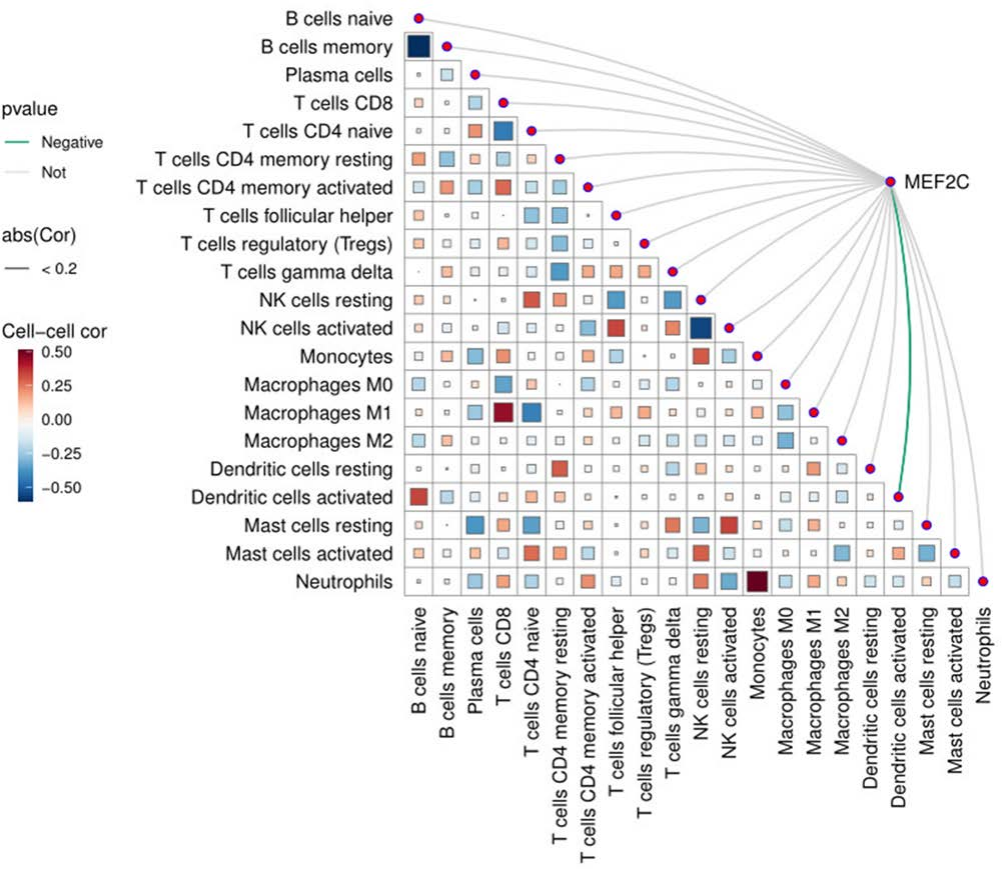
Heat map of *MEF2C* correlation with 22 immune cells.

The GSEA results suggest that *MEF2C* may be involved in the regulation of BPs and signaling pathways associated with immune infiltration. Therefore, this study further evaluated the level of immune-cell infiltration in OS samples versus normal control samples using the CIBERSORT algorithm and analyzed the differences in immune-cell distribution between the 2. The analysis showed that no significant difference in the degree of immune-cell infiltration was observed between OS and normal tissues (Fig. 6). In addition, we explored the correlation between *MEF2C* expression levels and 22 immune-cell subtypes and found that *MEF2C* was negatively correlated with activated dendritic cells (DCs) (Fig. 7).

### 3.6. PPI network analysis

**Figure 8.**
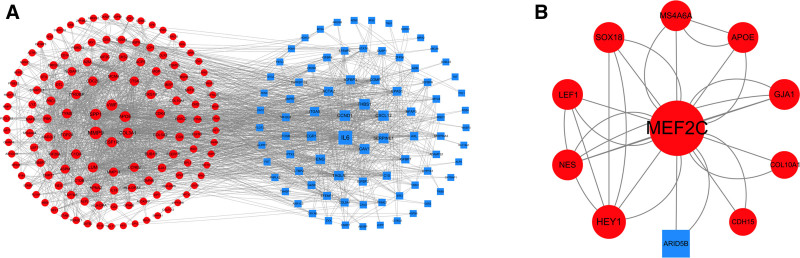
PPI network diagram. (A) Diagram of the PPI network of DEGs ; (B) Diagram of the PPI subnetwork centered on *MEF2C*. The red color in the figure is up-regulated genes, indicated by circles. Blue is down-regulated genes, represented by squares. Where the larger size of the font and graph represents the higher value of its degree, which is at the center of the network. DEGs = differentially expressed genes, PPI = protein–protein interaction.

To further explore the potential interactions of DEGs at the protein level, we constructed a PPI network of differential genes based on the STRING database and visualized and analyzed it with Cytoscape software. The results showed that several key transcription factors and signaling molecules, such as *IL-6*, *MMP9*, and *APOE*, had a high degree of connectivity (degree) in the network, suggesting that they may have a central role in the regulatory network. In addition, we further constructed a subnetwork graph centered on *MEF2C* and selected the top 10 interacting genes with the highest correlation with it for analysis. This subnetwork contains the following genes: *SOX18*, *MS4A6A*, *APOE*, *GJA1*, *COL10A1*, *CDH15*, *LEF1*, *NES*, *HEY1*, and *ARID5B*, which is down-regulated, and the rest are up-regulated. As shown in Figure 8.

### 3.7. Clinical relevance analysis

**Figure 9.**
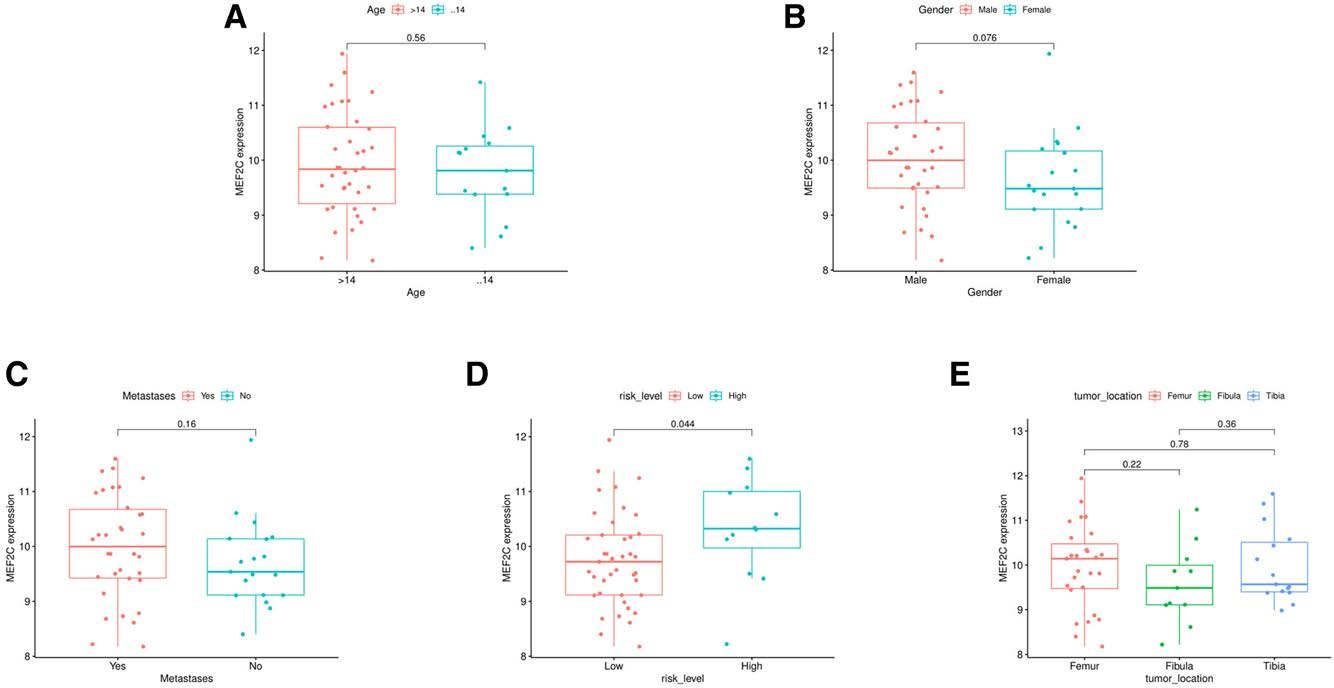
Analysis of clinical traits in *MEF2C* and OS patients. (A) Clinical correlation analysis between *MEF2C* and different ages; (B) Clinical correlation analysis between *MEF2C* and different genders; (C) Clinical correlation analysis between *MEF2C* and different metastatic statuses; (D) Clinical correlation analysis between *MEF2C* and different risk grades; (E) Clinical correlation analysis between *MEF2C* and different tumor sites. OS = osteosarcoma.

The results of the clinical correlation analysis showed that there was a significant difference in *MEF2C* expression between the high-risk grade group and the low-risk grade group, and no significant differences were found in the gender, age, metastatic status, and tumor site groups. As shown in Figure 9.

### 3.8. Survival analysis

**Figure 10.**
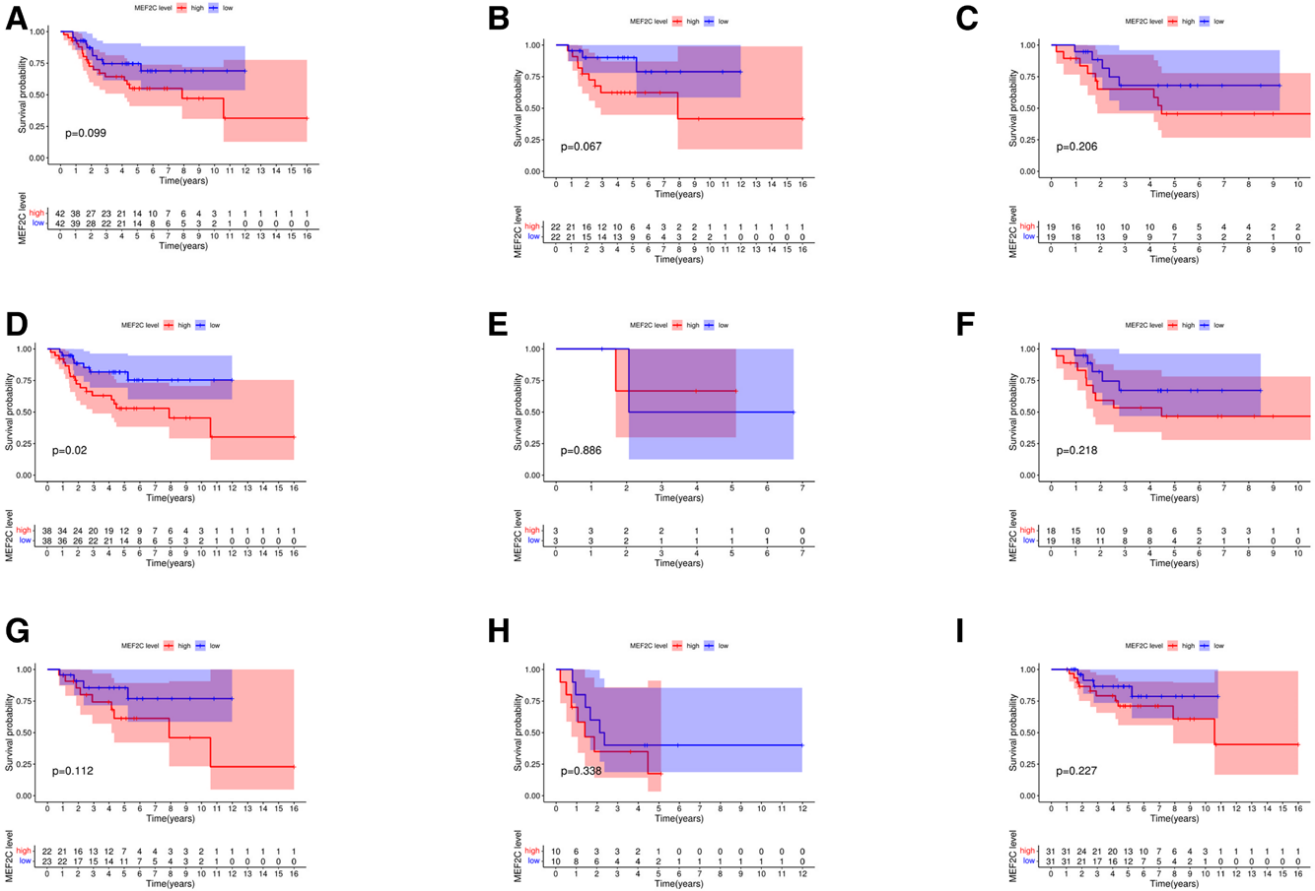
Relationship between *MEF2C* and overall survival of OS patients. Red color represents high gene expression group and blue color represents low gene expression group. The horizontal axis represents the overall survival time and the vertical axis represents the probability of survival. The following table lists the number of patients who remained alive at different time points from 0 to 16 yr. (A) relationship between *MEF2C* and overall survival of OS patients; (B and C) relationship between *MEF2C* and overall survival of OS patients aged ≤14 yr and >14 yr; (D and E) relationship between *MEF2C* and overall survival of OS patients with lower limb and upper limb; (F and G) relationship between *MEF2C* and overall survival of female and male OS patients; (H and I) relationship between *MEF2C* and overall survival of patients with metastasis and without metastasis in OS. OS = osteosarcoma.

In this study, the clinical and survival data of 87 OS patients were extracted from the TARGET database and further analyzed using the “survival” package to analyze the relationship between *MEF2C* gene expression levels and overall survival. Survival analysis showed no significant correlation between *MEF2C* expression level and overall survival (*P* > .05). To further explore its potential role in different clinical subgroups, we stratified the data by metastatic status (metastatic, nonmetastatic), gender (male, female), age (≤14 years, >14 years), and tumor site (upper extremity, lower extremity). The results showed that high *MEF2C* expression was significantly associated with overall survival in patients with lower-extremity OS (*P* = .02). As shown in Figure 10.

**Figure 11.**
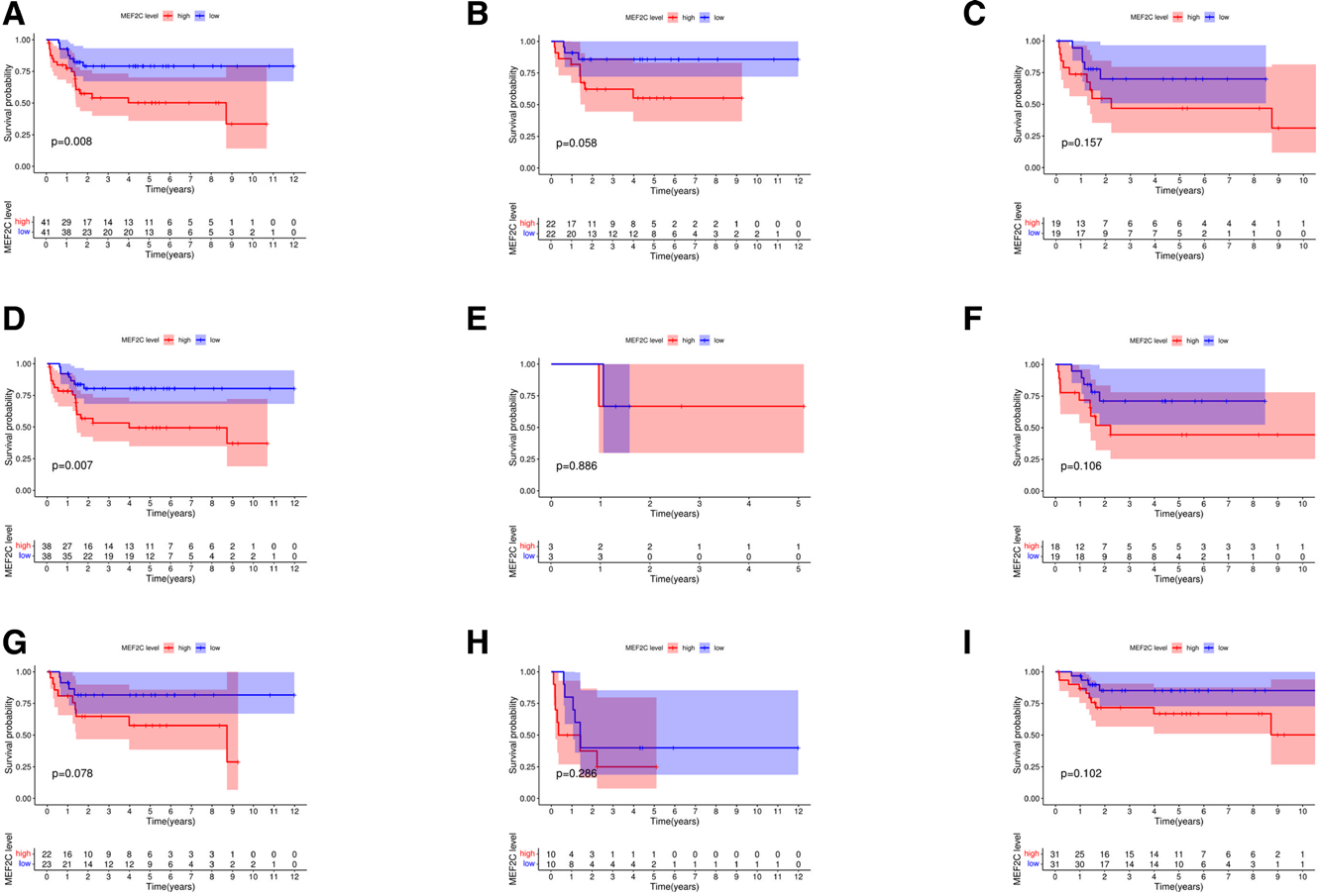
Relationship between *MEF2C* and EFS in OS patients. Red color represents the high gene expression group and blue color represents the low gene expression group. The horizontal axis represents the total event-free survival time and the vertical axis represents the time-free survival probability. The table below lists the number of patients who survived event-free in each year. (A) relationship between *MEF2C* versus EFS of total OS patients; (B and C) relationship between *MEF2C* versus EFS of OS patients aged ≤14 yr and >14 yr; (D and E) relationship between *MEF2C* versus EFS of OS patients with lower extremity and upper extremity; (F and G) relationship between *MEF2C* versus EFS of OS patients in females and males; and (H and I) relationship between *MEF2C* versus EFS of patients with metastasis of OS and those without metastasis. EFS = event-free survival, OS = osteosarcoma.

In addition, we analyzed the relationship between *MEF2C* expression levels and EFS. The results showed that *MEF2C* expression was significantly associated with EFS in patients with OS (*P* = .008), and further subgroup analysis revealed that *MEF2C* expression was significantly associated with EFS in patients with lower-extremity OS (*P* = .007). As shown in Figure 11.

### 3.9. Validation group analysis

**Figure 12.**
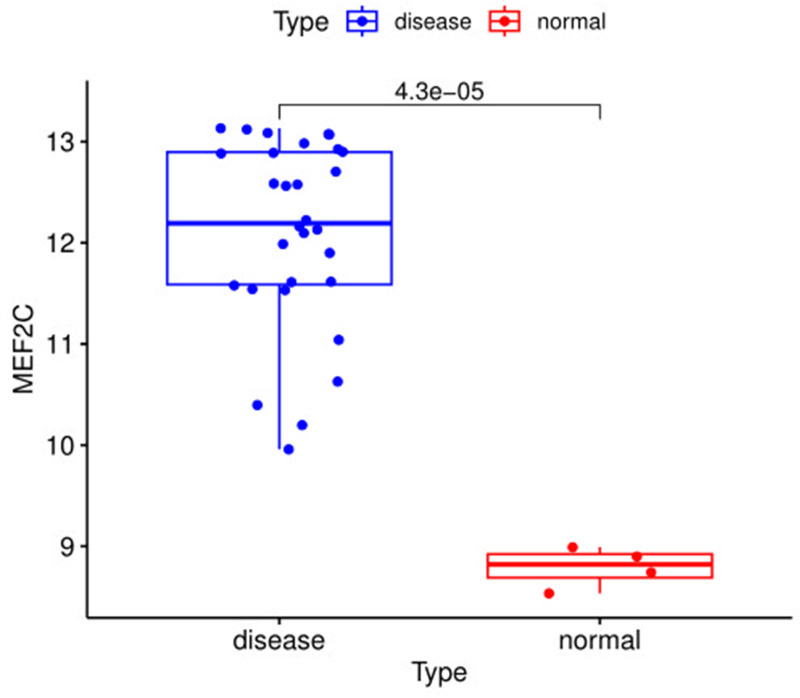
Expression of *MEF2C* in the validation group. Box plots of *MEF2C* expression levels in osteosarcoma and normal tissues. Disease: osteosarcoma group, normal: normal sample group.

The results of the validation group analysis showed that the expression of *MEF2C* was significantly increased in the OS samples compared to the control sample group (*P* < .001). As shown in Figure 12. The expression level of *MEF2C* in the validation group was consistent with the results of this study, which improved the reliability and robustness of the results of the MR analysis.

## 4. Discussion

OS, a highly malignant primary bone tumor prevalent in adolescents, is characterized by aggressive invasiveness, frequent recurrence, and a propensity for distant metastasis, all of which severely threaten patient survival.^[22,23]^ Despite advancements in survival rates through surgery and neoadjuvant chemotherapy, clinical treatment remains challenging due to the poorly understood molecular mechanisms underlying OS progression. In this study, we systematically analyzed 3 OS microarray datasets from the GEO database (117 OS samples and 27 normal tissues) using multiple bioinformatics approaches, including GO/KEGG enrichment analysis, MR analysis, immune-cell infiltration profiling, GSEA, PPI network analysis, and survival analysis. These analyses aimed to uncover molecular mechanisms of OS pathogenesis and identify genetic factors with therapeutic potential. Differential expression analysis identified a total of 269 DEGs associated with OS, and GO/KEGG enrichment analysis showed that these genes are mainly involved in functions and pathways related to extracellular matrix, bone morphogenesis, TGF-β signaling pathway and immunomodulation. Meanwhile, MR analysis was applied to evaluate genes closely associated with OS disease in DEGs, and 1 co-expression up-regulated gene (*MEF2C*) was obtained. MR analysis confirmed that increased *MEF2C* expression was associated with an increased risk of OS. The consistency of the results of the validation group analyses increased confidence in the association of *MEF2C* with OS disease. Survival analysis demonstrated that high *MEF2C* expression was significantly associated with shorter EFS in OS patients (*P* = .008). Notably, in lower limb OS cases, elevated *MEF2C* levels correlated with both reduced overall survival (*P* = .02) and EFS (*P* = .007), indicating its potential as a prognostic biomarker for disease progression.

Myocyte enhancer factor 2C (*MEF2C*), a *MADS-BOX* transcription factor encoded on chromosome 5q14.3, is involved in regulating cardiomyocyte differentiation, neurological development, and skeletal muscle formation.^[24–27]^ It also promotes cell proliferation and inhibits apoptosis.^[28,29]^ Previous studies have shown that *MEF2C* plays a role in a variety of cancers, and in lung adenocarcinoma, the level of *MEF2C* expression significantly correlates with the degree of immune infiltration in LUAD, directly influencing tumor immune escape.^[30]^ In pancreatic cancer it is associated with poor prognosis through enhanced TGF-β signaling.^[31]^ And cellular experimental studies have found that *MEF2C* is overexpressed in OS cells, which is consistent with the results of the present study.^[32]^ And this study also suggests that *MEF2C* serves as a direct target gene for microRNA-338-3p, and that their interaction in OS is associated with the promotion of cell proliferation and the inhibition of apoptosis, and that *MEF2C* may be a potential oncoprotein in the progression of OS. Animal studies by Sato et al demonstrated that *MEF2C* was able to act as a negative regulator of p53-responsive elements, and that p53 had an inhibitory effect on defective osteosarcoma cell lines.^[33,34]^ This suggests a regulatory interplay between *MEF2C* and p53 in driving OS progression. In this study, we found that *MEF2C* was highly expressed in OS by multi-omics analysis, and its increased expression was associated with an elevated risk of OS. Together, this evidence suggests a possible pro-cancer role of *MEF2C* in the development of OS.

Then, we further explored the activity level of *MEF2C* in OS by GSEA method, and our results indicated that *MEF2C* plays a multifaceted and complex role in OS. The expression level of *MEF2C* may be related to the development of the skeletal system, immune effector process, and other changes in biological functional activities. The active pathways in the *MEF2C* high-expression group suggested the possible presence of skeletal morphogenesis and TGF-β signaling pathways mediated by *MEF2C* regulation in OS, while the active pathways in the *MEF2C* low-expression group suggested the possible presence of immune effector processes mediated by *MEF2C* regulation in OS. Meanwhile, GO enrichment analysis showed that *MEF2C* was involved in the cellular response to TGF-β stimulation, and the overlap of this result with GSEA suggests that the TGF-β pathway plays a critical role in the promotion of OS development by *MEF2C*. Numerous studies have shown that the TGF-β signaling pathway plays a key role in tumor development.^[35,36]^ In recent years, the pro-cancer effect of this pathway in OS has also been confirmed by several studies.^[37,38]^ Therefore, we surmised that *MEF2C* in OS may influence the development of OS by regulating the TGF-β signaling pathway.

In addition to exploring the specific effects of genes on OS, we extended our study to the immune level of OS. We analyzed the level of immune-cell infiltration using the CIBERSORT algorithm. The results revealed that no significant difference was found in the level of immune-cell infiltration between osteosarcoma and normal tissue samples. We further analyzed the correlation between *MEF2C* and 22 immune-cell subpopulations and showed that *MEF2C* was negatively correlated with DCs activated, *MEF2C* was negatively associated with regulatory T cells, follicular helper T cells, type 1 T helper cells, macrophages, MDSC, natural killer cells, activated DCs, and CD8 + T cells in the study of Guo et al.^[39]^ The concordance between the 2 studies reinforces the strong association between this gene and activated DCs in OS. And previous studies have shown that mature DCs are capable of initiating the antitumor response of helper T cells-1 through the production of pro-inflammatory cytokines.^[40]^ When *MEF2C* is highly expressed in OS, the number of activated DCs is subsequently reduced, which in turn may affect the antitumor response of immune cells. Enrichment analysis showed that *MEF2C* involves the TGFβ signaling pathway, while the study of Travis et al concluded that TGF-β inhibits DC differentiation and activation.^[41]^ Based on these findings, we suggest that *MEF2C* overexpression may promote TGF-β production and inhibit DC activation, which in turn exerts a pro-cancer effect.

PPI network analysis identified genes such as *IL-6* and *CXCL12* as down-regulated center target genes and genes such as *MMP9* and *APOE* as up-regulated target genes. Of note, Apolipoprotein E (*APOE*) has been strongly associated with a number of cancers, such as lung cancer, hepatocellular carcinoma, and pancreatic ductal adenocarcinoma.^[42–44]^ It has been shown in animal studies that elevated *APOE* levels are associated with immunosuppression, and higher serum *APOE* levels are associated with lower patient survival.^[44]^ Studies in mouse models of pancreatic ductal adenocarcinoma have further demonstrated that *APOE -/*- mice have elevated levels of intratumor CD8 + T cells compared to wild-type mice. Subsequently, we further constructed a subnetwork graph centered on *MEF2C* and selected the top 10 interacting genes with the highest correlation with it for analysis, and *APOE* was used as an up-regulated target gene in the subnetwork. In addition, it has been shown that there is a direct interaction between *MEF2C* and *SOX18*, that *MEF2C* enhances the transcriptional activity of *SOX18*, and that high expression of *SOX18* plays an important role in the proliferation, apoptosis, migration, and invasion of OS cells.^[45,46]^ This suggests that the 2 may have a synergistic effect in regulating the expression of vascular-related genes, which may play an important role in angiogenesis, tumor invasion, and metastasis of osteosarcoma, especially in the early stages of vascular and lymphatic vessel formation.

Finally, we used a validation group for differential expression analysis, and the results were consistent with the MR analysis, which confirms the reliability of our findings. High expression of *MEF2C* may contribute to the development of OS by affecting processes such as bone morphogenesis, extracellular matrix remodeling, and immune immunomodulation. However, all these possibilities need to be validated by further experiments to elucidate the specific role of *MEF2C* in OS. However, this study has some limitations. First, only a single bioinformatics analysis was performed in this study, and in vitro and in vivo studies were lacking to validate the results. Second, there may be potential selection bias due to limited sample sources and analysis methods. In the future, we will conduct more in-depth clinical studies and laboratory validation. Certainly, the potential therapeutic targets identified in the latest study deserve further attention. For example, Zhang et al found that *USP22* was highly expressed in OS and was associated with poor prognosis in OS patients, and found that down-regulation of *USP22* inhibited glycolysis and growth of OS cells by in vitro experiments.^[47]^

## 5. Conclusion

In summary, this study analyzed OS in detail using multiple bioinformatics methods and identified key genes, revealing that *MEF2C* may be involved in the development of OS by affecting related functions and pathways such as extracellular matrix and immunomodulation. These findings suggest that *MEF2C* may be a potential prognostic marker for OS and provide direction for exploring new potential therapeutic intervention strategies. However, these results require further validation and consideration of the limitations of data selection and analysis techniques themselves. Overall, this study contributes to a deeper understanding of OS and demonstrates the need for targeted molecular and immunological approaches in future therapeutic and research strategies.

## Acknowledgments

We express our gratitude for the valuable data resources provided by the researchers and the databases.

## Author contributions

**Conceptualization:** Zhihui Zheng, Zhicheng Liao, Liang Pang.

**Data curation:** Zhihui Zheng.

**Methodology:** Zhicheng Liao, Liang Pang.

**Project administration:** Zhihui Zheng, Qinglai Wang.

**Software:** Zhihui Zheng.

**Supervision:** Pengcheng Jia, Qinglai Wang.

**Visualization:** Zhouhengte Xu, Yibo Zhu.

**Writing – original draft:** Zhihui Zheng.

**Writing – review & editing:** Zhicheng Liao, Zhouhengte Xu, Yibo Zhu, Pengcheng Jia, Qinglai Wang.

## Supplementary Material


