# Supplementary figures and images for: MEF2C is a potential prognostic biomarker for osteosarcoma
Source: Medicine (Baltimore). 2025 Sep 5;104(36):e44313. doi: 10.1097/MD.0000000000044313 (PMC12419286; doi:10.1097/MD.0000000000044313)

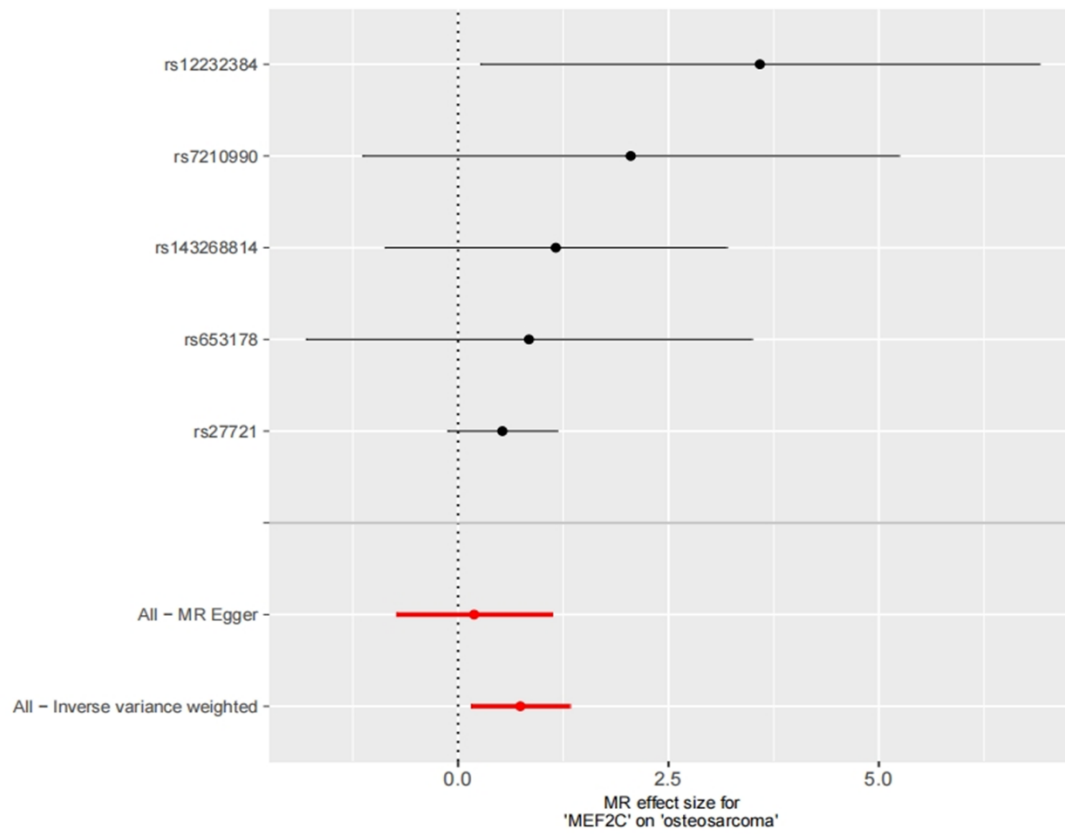

MR Method

- Inverse variance weighted
- MR Egger

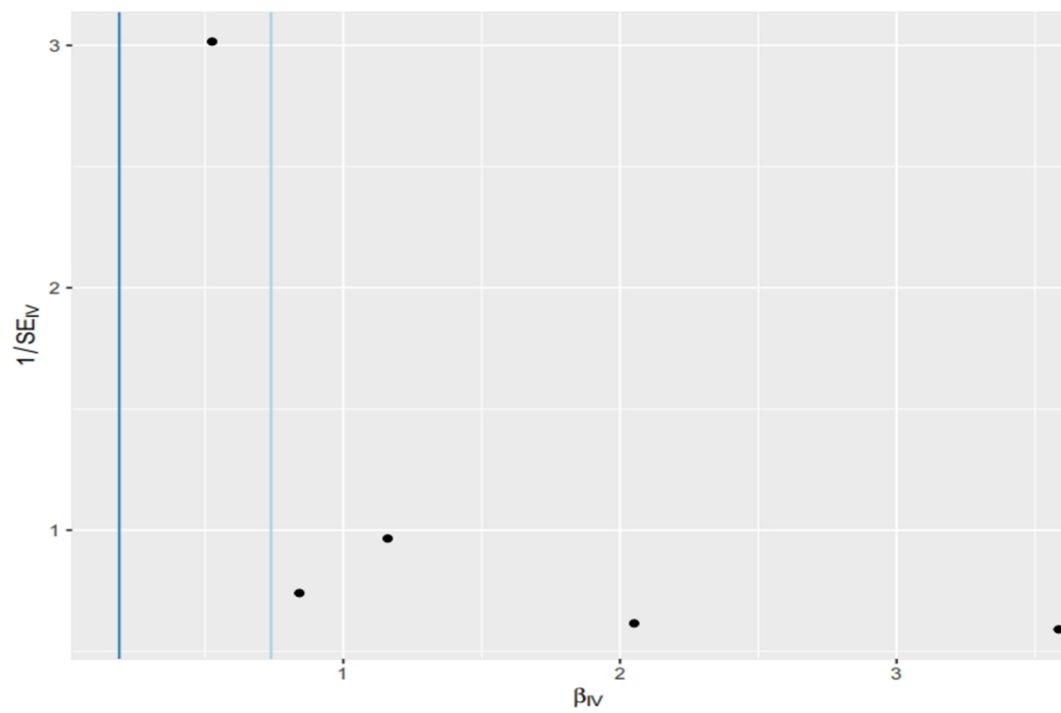

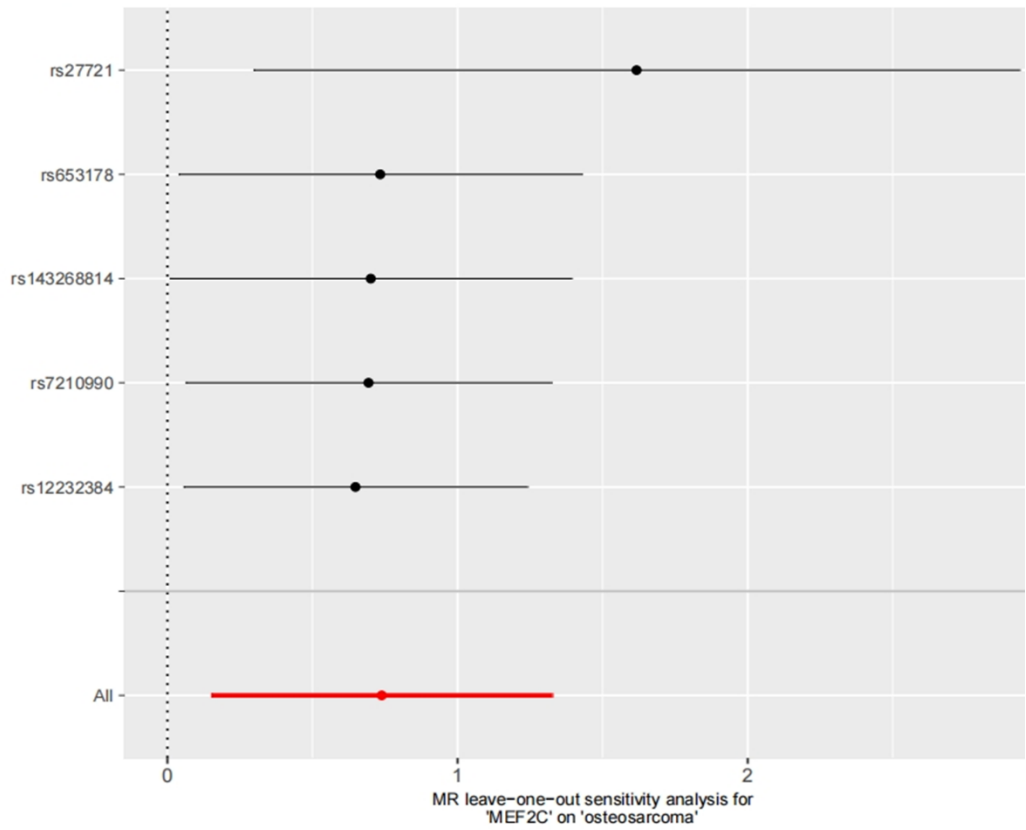

#### MR Test

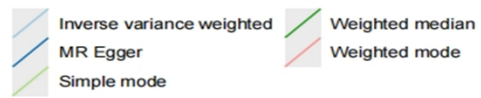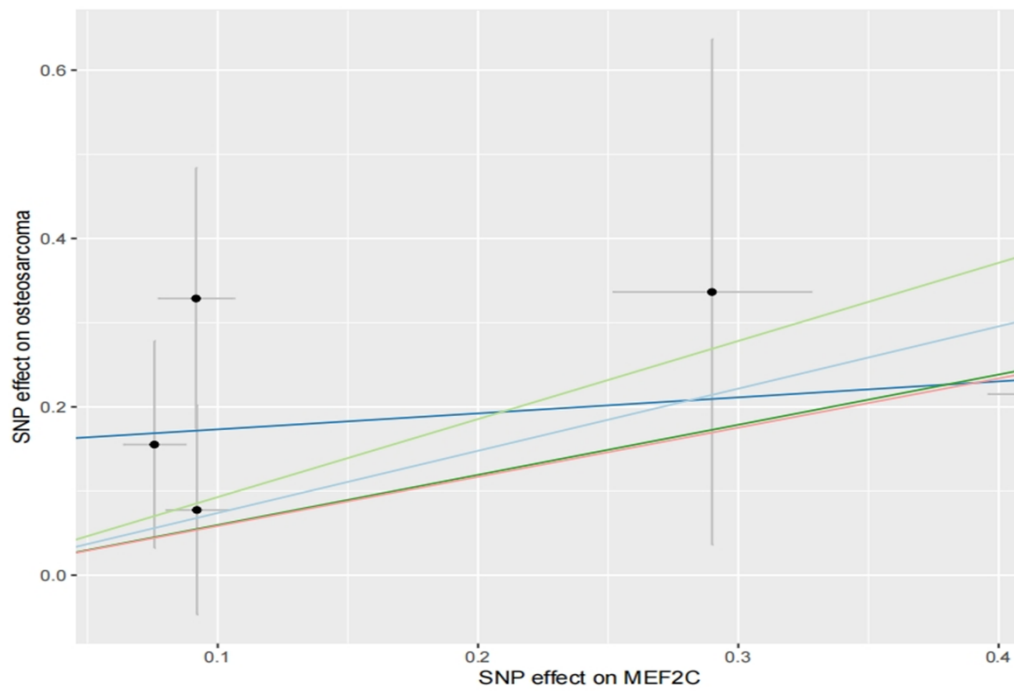

Supplement: Supplementary file 2 [file medi-104-e44313-s002.pdf]
